# Education Reform and Quality Training of Music Majors From the Perspective of Entrepreneurial Education

**DOI:** 10.3389/fpsyg.2021.749701

**Published:** 2021-12-24

**Authors:** Yue Jiang

**Affiliations:** Shenyang Conservatory of Music, Shenyang, China

**Keywords:** entrepreneurship education, entrepreneurship, music major, entrepreneurial situation, reform

## Abstract

Art major graduates are facing more severe employment pressure. Based on entrepreneurial education, the reform of entrepreneurship education and quality training of music majors in colleges and universities are analyzed. First, the relevant theories of entrepreneurship education are introduced and the advantages of advanced entrepreneurship education in foreign countries are analyzed. Second, the music majors in a university are selected as the subjects to analyze the current situation of entrepreneurship of music majors. Finally, new strategies are put forward for the reform of entrepreneurship education of music majors. The research shows that the number of music majors who are very interested in entrepreneurship accounts for 22.2%. In terms of the music majors’ understanding of entrepreneurship policies, the proportion of students with less understanding accounts for 40.1%. As for the ways of music majors to acquire entrepreneurship knowledge, 8.00% usually turn to books and networks. Music majors hope to get support from the government and schools in the cultivation of entrepreneurship. In entrepreneurship, music majors who intend to start a business according to their interests account for 40.8%. The variance of eigenvalues of the four main factors is 19.49, 12.96, 10.75, and 8.39%, respectively, and their contribution value is 51.58%. The four research aspects of entrepreneurship education are music majors’ entrepreneurship policy, entrepreneurship desire, and entrepreneurship quality and entrepreneurship education practice. The entrepreneurship education system is to arouse interest, improve entrepreneurial ability, and form entrepreneurial personality. This paper proposes good entrepreneurship education strategies.

## Introduction

Since the twenty-first century, there are more and more entrepreneurs worldwide with the rapid development of the economy, and the number of young entrepreneurs also increase significantly (Helle et al., 2020; Botha et al., 2021; Shekhar and Huang, 2021). In this case, China has strengthened the cultivation of college students’ entrepreneurial ability accordingly. Entrepreneurship education aims to cultivate entrepreneurs (Ayob, 2021), including improving the entrepreneurial ability of the educated by strengthening their entrepreneurial awareness, entrepreneurial skills, and entrepreneurial thinking (Gloria et al., 2019; Mir and Alarifi, 2021; Nicotra et al., 2021). Due to the severe employment environment in China, entrepreneurship education can play a certain role in alleviating job hunting pressure. In addition, entrepreneurship education also positively accelerate the transformation of university knowledge level, promoting the cooperation between schools and employers and improving the overall national innovation ability in China (Yuan et al., 2020; Hsu and Pivec, 2021; Lambert and Rennie, 2021). Because of this, China has introduced many policies to encourage college graduates to start a business this year, and many colleges and universities begin to increase investment in entrepreneurship education. However, compared with the 20% self-employment rate of college graduates in developed countries like Europe and America, the number of college graduates in China only accounts for 1% of the total number (Helen et al., 2021; Zaring et al., 2021). The low rate of entrepreneurship shows the poor effect of entrepreneurship education in colleges and universities in China. Therefore, this study aims to optimize the entrepreneurship education of college graduates.

The overall self-employment rate of Chinese college graduates is low, and art graduates face the same challenge. In recent years, the number of music graduates in colleges and universities has surged, and the number of posts corresponding to music graduates is less. The employment difficulty of music graduates becomes increasingly prominent (Teresa, 2019; Chang et al., 2020). In this case, many music graduates realize their values by establishing music counseling institutions, personal music rooms, and cultural performance companies (Yang, 2018; Du, 2020). However, most graduates end up with entrepreneurial failure, and only a small part of them succeed in entrepreneurship due to the lack of scientific theoretical guidance (Sabahat, 2019). At present, entrepreneurship education in colleges and universities is the same for all majors, and most colleges and universities still adopt the same entrepreneurship education training system for all majors, which leads to poor the effect of entrepreneurship education (Koops and Kuebel, 2019). The focus of college entrepreneurship education should be different according to the characteristics of different majors, ultimately creating a suitable entrepreneurship training system for different majors.

Due to the severe situation of the employment market, employment difficulty has become one of the problems faced by art graduates in colleges and universities. Therefore, entrepreneurship becomes one of the main choices of many art majors. This study takes music majors in a university as an example to study and analyze the education reform and quality training of music majors based on entrepreneurship education, aiming to explore effective methods to guide music majors’ entrepreneurship. First, the literature method and comparative analysis method are used to summarize the research in China and foreign countries, and the related concepts involved in this study are defined and introduced. The questionnaire survey and factor analysis method are used to analyze the current situation of music majors’ entrepreneurship, and the main factors affecting the entrepreneurial intention of music graduates are found out. On this basis, the entrepreneurship education and training system for music majors is established. In previous studies, there are few studies on entrepreneurship of art majors. The innovation of this study is to establish an entrepreneurship education and training system suitable for music majors. The research content promotes the reform and development of higher education.

### Literature Review and Analysis of Entrepreneurship Education Reform

#### Literature Review

**FIGURE 1:**
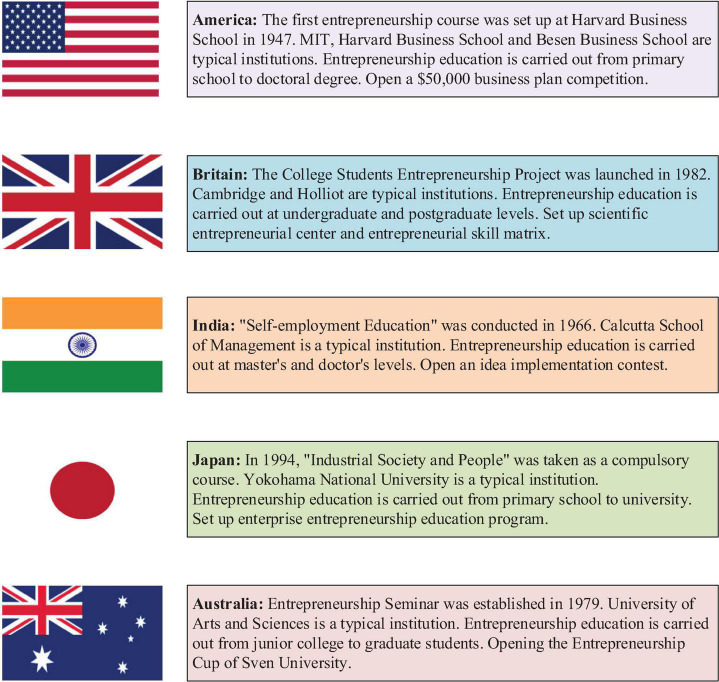
Excellent entrepreneurship education in foreign countries.

Entrepreneurship education in foreign countries starts in 1940. Harvard University in the United States first opened entrepreneurship education courses and published *Entrepreneurship History Exploration* in the late 1940s. This book is the first book describing entrepreneurs (Hildesheim and Sonntag, 2020; Park et al., 2020). The entrepreneurship education program at Harvard University has achieved good results (Suleman and Figueiredo, 2020). Since 1980, many universities in the United States offer entrepreneurship education courses for all students and increase the investment in entrepreneurship research (Smolarek and Scrivener, 2019; Karabassova, 2021). Entrepreneurship education in the United States covers a wide range, and entrepreneurship education opens from primary school to doctorate education. The development of entrepreneurship education promotes the economic development of the United States. American entrepreneurship education ranks the leading position in the world. Entrepreneurship education in Germany begins at the end of the twentieth century, and professional entrepreneurship education and training courses are set up at the beginning of the twenty-first century. The entrepreneurial rate of graduates reached up to 30% (Hu and Hu, 2021). Nanyang Polytechnic University in Singapore is one of the most perfect schools in the implementation of entrepreneurship education in the Asia-Pacific region. The courses offered are more practical, and the courses mainly focus on solving the problems encountered by students in the process of entrepreneurship (Rudzani, 2019). The details of excellent entrepreneurship education in foreign countries are shown in Figure 1.

Figure 1 shows that entrepreneurship education in the UK began in the 1980s, and Cambridge University in the UK took the lead in launching the “College Students’ Entrepreneurship” project, which carried out entrepreneurship education among undergraduates and postgraduates. Entrepreneurship education in Australia is almost synchronous with that in Britain (Maximilian and Viola, 2021). Japan opened entrepreneurship education courses at the end of the twentieth century, which are included in compulsory courses (Angelina and Nceba, 2017; Mohammad, 2019). India began to develop entrepreneurship education in 1966, and the training subjects are mainly masters and doctors (Madziva and Thondhlana, 2017; Sergeeva et al., 2021). South Korea has established the alumni association of college students’ entrepreneurship to provide technical references for college students’ entrepreneurship (Ma et al., 2021). Many advanced research results are worthy of further study and reference.

Entrepreneurship education in China began in the 1990s, and the related research on entrepreneurship education was carried out in Liaoning, Sichuan, and Beijing. Entrepreneurship education develops rapidly in the early twenty-first century. The research on entrepreneurship education in China mainly focuses on its problems, contents, and policies. The current problems of entrepreneurship education in China include that entrepreneurship education is not integrated with the overall school education system, the concept of entrepreneurship education is out of track with the times, the lack of entrepreneurship education resources and the practice of entrepreneurship education is not strong. The curriculum system of entrepreneurship education in China mainly includes activity courses, subject courses, environmental courses, and practical courses. The research on the model and approach of entrepreneurship education shows the characteristics of the times and focuses more on team learning. The research on the specific strategies of Chinese college students’ entrepreneurship education mainly analyzes the differences between the policies of China and foreign countries. China has fewer policies on entrepreneurship education than foreign countries.

After the previous studies are reviewed, it is found that there is little theoretical research and practical summary of entrepreneurship education in China, and the theoretical research on entrepreneurship education lacks further conclusions, quantitative analysis, and empirical research. The practice of entrepreneurship education in foreign countries is strong, and the empirical study of entrepreneurship education in China is still in its infancy. The management of entrepreneurship education in China is not highly concerned, and the entrepreneurial activities of colleges and universities are not supported. Graduates start their businesses in the absence of entrepreneurial ability, which causes many entrepreneurial failures. Compared with the mature entrepreneurial environment in foreign countries, the public opinion on college students’ entrepreneurship in China is not optimistic. In view of the above reasons, China’s entrepreneurship education still has a long way to go.

#### Relevant Concepts of Entrepreneurship Education

In the studies in China and foreign countries, there are many explanations for the concept of “entrepreneurship.” The mainstream concept is that entrepreneurship is a kind of behavior mode and thinking mode, through which people create economic benefits. This statement is used to define “entrepreneurship” here. Entrepreneurship education is the education of entrepreneurship, the education for developing the basic quality of college students’ entrepreneurship, and the education for cultivating college students’ entrepreneurial skills. This study defines entrepreneurship education as an educational model that combines social practice with classroom education to systematically cultivate college students’ self-employment ability.

**FIGURE 2:**
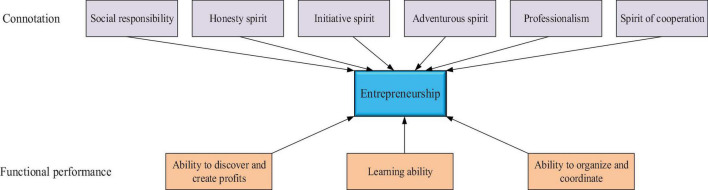
The connotation of entrepreneurship.

Entrepreneurship is an innovative spirit, and it also includes the spirit of cooperation, dedication, adventure, honesty, and social responsibility. The specific connotation of entrepreneurship is shown in Figure 2.

Figure 2 shows the components of entrepreneurship, namely the spirit of cooperation, dedication, adventure, honesty, and social responsibility, as well as the three functions. Among them, innovation spirit is the core of entrepreneurship, and it requires entrepreneurs to have the keen analytical ability, insight ability, and value judgment ability, which is consistent with the conclusion that entrepreneurs tend to have higher adaptability (Chen, 2019). The spirits of dedication and cooperation are the pillar of entrepreneurship (Qian et al., 2018). Adventurous spirit shows entrepreneurs’ courage to try (Yuan and Wu, 2020). Honesty spirit is the cornerstone of entrepreneurship (Wu and Wu, 2017). Learning ability, organizational coordination ability, and ability to discover and create profits ultimately help enterprises to create the most profits by shaping different abilities of entrepreneurs.

In short, entrepreneurship can be acquired through entrepreneurship education. Rogoza et al. (2018) found that personality traits play an important role in competition (Rogoza et al., 2018). The connotation of entrepreneurial spirit is consistent with the objectives of entrepreneurship education. The successful experience of the developed countries like the United States shows that the ultimate goal of entrepreneurship education is to cultivate positive and adventurous entrepreneurial talents. In addition, entrepreneurship education should pay more attention to the personality cultivation of college students. If college students have the spirit of entrepreneurship, they will succeed in starting a business.

#### Analysis of the Reform and Quality Training of Entrepreneurship Education

Entrepreneurship education in China has made some achievements. It is set as a compulsory course in colleges and universities. The content of the course opened in entrepreneurship education is no longer theoretical learning, as well as practice teaching. However, there are still some problems in entrepreneurship education in colleges and universities in China. The biggest problem is that the concept of entrepreneurship education is backward; most college students do not take entrepreneurship as the first choice, and the weak foundation of social entrepreneurship is also one of the problems. Due to the influence of Chinese traditional culture, Chinese people lack the spirit of adventure as a whole and think that only employment is the only way after graduation.

Entrepreneurship of art students has strong professional characteristics. For example, music majors tend to have a strong desire to express themselves and are more suitable for self-employment, rather than engage in relatively boring work. In addition, the cost of entrepreneurship for music majors is lower. Entrepreneurial majors can start their businesses only if they have professional skills. As mentioned above, there are many ways for music majors to start a business, like setting up training institutions.

The entrepreneurship education system in the United States is relatively perfect. Entrepreneurship education encourages students to start their businesses, pays more attention to teaching students following their aptitude, focuses on the practice of entrepreneurship, and strives for social resources. In addition, most professors of entrepreneurship education in the United States have entrepreneurship, so they have strong entrepreneurial guidance ability (Zheng et al., 2018; Wu et al., 2019). Singapore is the most successful country in implementing entrepreneurship education in the Asia-Pacific region. The Nanyang University of Technology has established entrepreneurship education as an independent major. The implementation of entrepreneurship education in France focuses more on the cultivation of personality (Wu and Song, 2019; Wu W. et al., 2020; Wu Y. J. et al., 2020).

**FIGURE 3:**
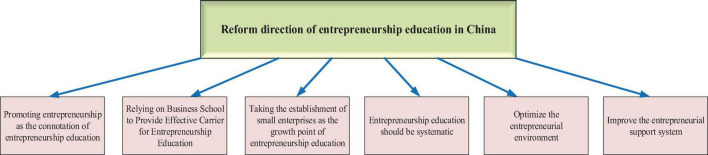
Reform direction of entrepreneurship education in China.

Based on the above, the suggestions on the reform direction of entrepreneurship education in China are shown in Figure 3.

Figure 3 shows that the reform of china’s entrepreneurship education should pay more attention to the cultivation of entrepreneurship. Entrepreneurial success is closely related to the personality traits of entrepreneurs. Entrepreneurship education in western countries attaches importance to the cultivation of entrepreneurship and achieves good results. Teachers should hire teachers with entrepreneurial experience who can make a key contribution to entrepreneurship education. It is necessary to set up small enterprises as the curriculum objective of entrepreneurship education, build an entrepreneurship system, optimize the social environment of entrepreneurship, gain government support and create a good social environment for entrepreneurs.

#### Analysis on the Current Situation of Music Majors’ Entrepreneurship

The case study is conducted by a questionnaire survey on the music majors in a university and the students’ views on entrepreneurship education are mainly studied. The influencing factors of the entrepreneurial intention of the music majors are explored through factor analysis.

#### Questionnaire Design

The music majors in a university are taken as the research subjects, and the objectives of the major are to cultivate music professionals with perfect theoretical knowledge, practical ability, music appreciation ability, and music teaching ability. The cultivation of professional teachers and curriculum construction of entrepreneurship education has made some achievements.

**FIGURE 4:**
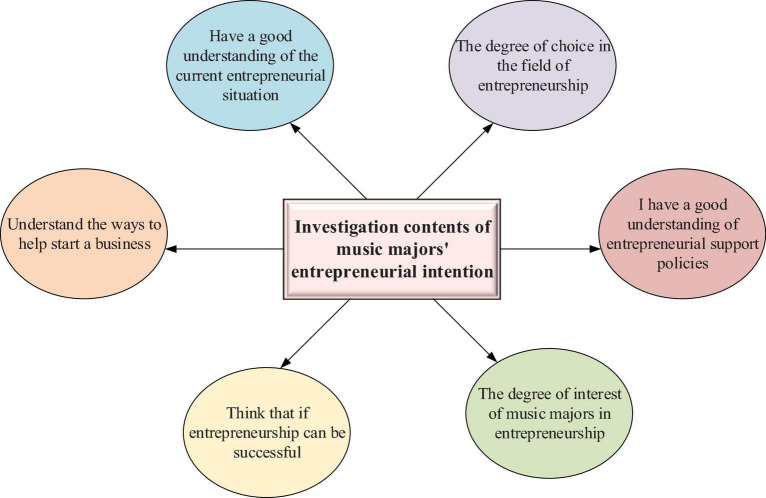
Basic questions in the questionnaire.

In order to fully reflect the authenticity of the questionnaire, the selected subjects are Piano Majors and non-Piano Majors. The total number of respondents is 200, including 100 in school A and 100 in school B. a total of 200 questionnaires were distributed, 200 were recovered and 200 were valid. The effective rate of the questionnaire is 100%. The questionnaire consists of two parts: The first part includes the basic questions, and the second is the survey on students’ entrepreneurial intention. The statistics on the basic questions of this questionnaire are conducted manually. The statistical results of the first part are shown in Figure 4.

Figure 4 shows the survey on the entrepreneurial intention of music majors, including their interest in entrepreneurship, the possibility of success in entrepreneurship, the understanding of the current entrepreneurial situation, the understanding of entrepreneurial support policies, the understanding of ways to help entrepreneurship, and the choice in the field of entrepreneurship. The setting of basic questions lays the foundation for the study of music majors’ entrepreneurial cognition.

The options to the 6 basic questions in the questionnaire are numbered from 1 to 5. They correspond to thoroughly understand, relatively understand, understand, understand a little, totally understand, respectively, to the questions of their interest in entrepreneurship, the possibility of success in entrepreneurship, the understanding of the current entrepreneurial situation, the understanding of entrepreneurial support policies. The options to the question of whether understand the ways to help start a business are numbered from 1 to 5, respectively, and they are the relevant policy, school courses, family support, participation in social activities, and books and network resources. The options to the choice in the fields of entrepreneurship are numbered from 1 to 5, respectively, and they are the field related to their majors, the field of their interests, the popular field, the field of less risk, and others. Table 1 shows the gender of the respondents.

**TABLE 1:** Gender of respondents.

Gender	University A	University B
Male	45	48
Female	55	52

#### Factor Analysis

The entrepreneurial rate and success rates of Chinese college students are low. The influencing factors of the entrepreneurial intention of music majors are studied, which lays the foundation for the development of entrepreneurship education mode suitable for music majors. The evaluation index system of college students’ entrepreneurial intention is constructed. The index system contains a total of 17 evaluation indexes that are basically consistent with the questionnaire topics. Here the Likert scale is used for scoring the data of the questionnaire. The options of the evaluation index are “totally disagree, disagree, neutral, agree, and totally agree,” expressed by 1–5. The analytical tools are Statistical Product and Service Solutions 25.0.

The second part of the questionnaire is the specific index settings, as shown in Table 1.

Table 1 shows that the content of the evaluation index system of entrepreneurial intention is designed through the collection of media reports, entrepreneurial case analysis, and interview records of music majors.

#### Preparation for Factor Analysis

SPSS 25.0 is used to analyze the correlation between the above influencing factors. Bartlett spherical test and Kaiser-Meyer-Olkin test are used to test the data. The results show that the result of the Bartlett spherical test is 1121.80, and the significance level is 0.0010.6, indicating that the sample can conduct factor analysis. Table 2 shows the evaluation index system of entrepreneurial intent.

**TABLE 2:** Evaluation index system of entrepreneurial intention.

General goal	Specific evaluation index	Grade
Personal entrepreneurial	Always try to constantly improve and breakthrough yourself	I1
willingness	A man who dares to take risks	I2
	The government has introduced a large number of policies conducive to entrepreneurship	I3
	Industry rivals have strong competitiveness	I4
	The school has relevant policies to support entrepreneurship	I5
	Have a lot of workplace internship experience	I6
	Master some economic and management knowledge	I7
	Most of my family and friends have entrepreneurial experience	I8
	If I start a business, my parents and relatives will support me	I9
	The local government has certain support policies for entrepreneurship	I10
	Independent and confident, try to grasp and solve some things	I11
	There are often some strange ideas	I12
	Successful entrepreneurial deeds will affect my choice	I13
	I hope to be recognized by my friends around me	I14
	Have a strong desire to achieve personal career success	I15
	I hope to create and accumulate more wealth	I16
	I hope to be a person who contributes to the country and society	I17

The principal component analysis is used to extract the common factors of 17 indexes. The characterization values included the contribution rate, the eigenvalue, and the cumulative contribution rate, and the number of the factors is set to 4. The eigenvalues of each index are compared in the “Result” section. SPSS software eventually produces the main influencing factor matrix, and the variance maximization orthogonal rotation method is used to rotate the main factors, obtaining the factor load value after rotation. If the factor load value is greater than 0.5, the corresponding index has obvious significance.

### Analysis on the Current Situation of Music Majors’ Entrepreneurship

#### Statistical Results of Basic Questions in the Questionnaire

**FIGURE 5:**
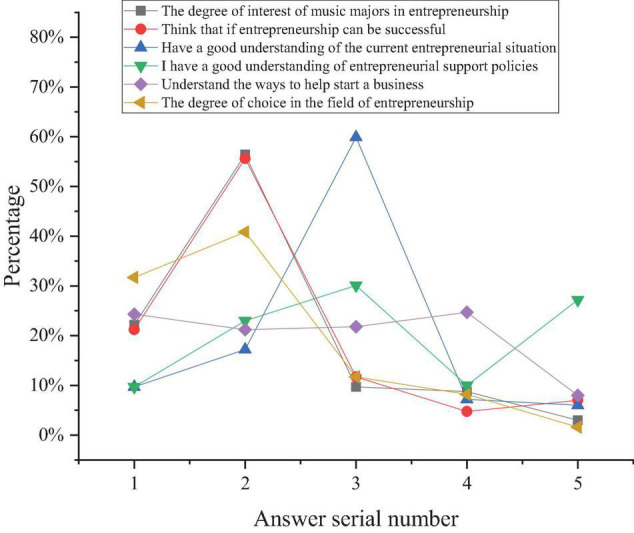
Results of the entrepreneurial intention of music majors.

The results of music majors’ entrepreneurial intentions are shown in Figure 5.

Figure 5 shows that the number of music majors who are very interested in entrepreneurship accounts for 22.2%, and the number of students interested in entrepreneurship accounts for 56.4%; most music majors are still interested in entrepreneurship, which is related to the current employment trend and school entrepreneurship education. The number of music majors who think that they can succeed in entrepreneurship accounts for 21.2%, and the proportion of the students who think that they can succeed in entrepreneurship is 55.6%, indicating that the majority of music majors have certain entrepreneurial confidence and that entrepreneurship education plays a certain role in entrepreneurial success. The proportion of the students with a very positive attitude to the current entrepreneurial situation is 9.7%, and the proportion of the students with a generally positive attitude is 17.2%. The proportion of the students with opportunities is the highest, reaching 59.9%. This shows that music majors have a positive attitude to the current entrepreneurial situation, which lays a foundation for entrepreneurship education and quality training. In music majors’ understanding of entrepreneurial support policies, the proportion of students with less understanding is 40.1%, the proportion of students who do not know is 27.2%, and the proportion of students with more understanding is the least, which is 9.7%. This shows that some students are not interested in entrepreneurship and remain indifferent to entrepreneurship education. In terms of the ways for music college students to obtain entrepreneurial assistance, 8.00% of the students usually get help through books and networks, which is the lowest proportion, and the proportion of other ways is basically the same. Among them, the number of students who get help from social activities is the largest, reaching 24.7%, indicating that music students want to get entrepreneurial support from the government and schools. As for the choice of the field of entrepreneurship, the highest proportion of the students choose the field according to their interest, accounting for 40.8%, followed by according to their majors, accounting for 37.7%, which is consistent with the overall entrepreneurial situation of college students. The above data analysis suggests that music majors have great intention to start a business, and music majors have a positive attitude toward entrepreneurship. However, some survey results also show that students’ entrepreneurial intention is positively correlated with entrepreneurship education, and government support is positively correlated with music majors’ entrepreneurial intention. In addition, the survey results show that, for music majors, participating in social activities can cultivate entrepreneurial intention.

#### Evaluation Index System of Entrepreneurial Intention

**FIGURE 6:**
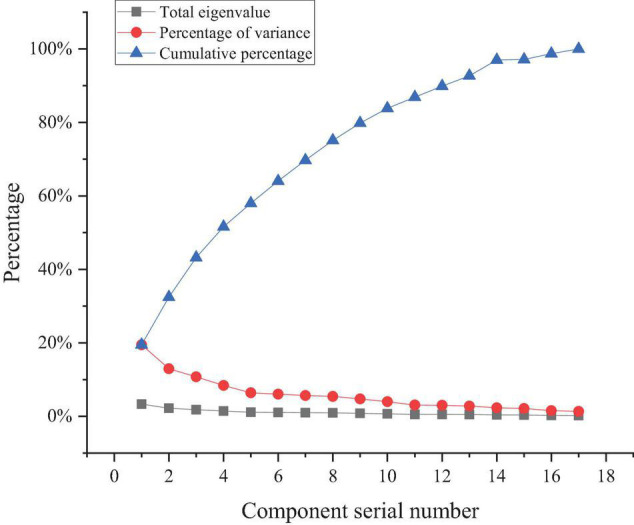
Comparison results of the eigenvalues of 17 indexes.

The comparison results of the eigenvalues of 17 indexes are shown in Figure 6.

Figure 6 shows that the variances of the eigenvalues of the first four factors are 19.49, 12.96, 10.75, and 8.39%, respectively. The cumulative contribution of the first four factors reaches 51.58%, indicating that these four factors can reflect the information of the original variable of 51.58%. These four factors are selected as the main factors affecting the entrepreneurial intention of music majors. The follow-up results analyze the specific indexes and significance of the 17 indexes corresponding to the four factors. The survey results in Figure 6 show that the main intention of music majors to start a business has four determinants, namely: “I14, I hope my friends around can recognize me”;“I15 I have a strong desire to achieve personal career success”; “I16 I hope to create and accumulate more wealth”; The four factors of “I17 I hope to be a person who contributes to the country and society” have a great impact on the entrepreneurship of music majors.

#### Orthogonal Rotation Results of the Principal Component Matrix and Factor Variance Maximization

**FIGURE 7:**
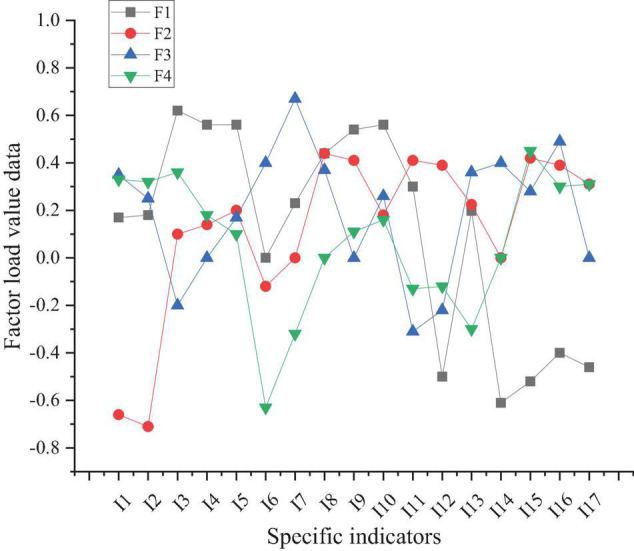
Load values of the four principal component factors (F1, F2, F3, F4 represent four principal component factors).

The load values of the four principal component factors are shown in Figure 7.

Figure 7 shows that the specific load values of the four main components in the 17 indicators are widely distributed. The specific significance of factor 1 corresponds to indicators I3, I4, I5, I8, I9, and I10, but the corresponding indicators of the other factors are not clear. Therefore, the maximum rotation of the variance is carried out to obtain the corresponding indicators of the four factors more clearly and optimize the content of Figure 7.

**FIGURE 8:**
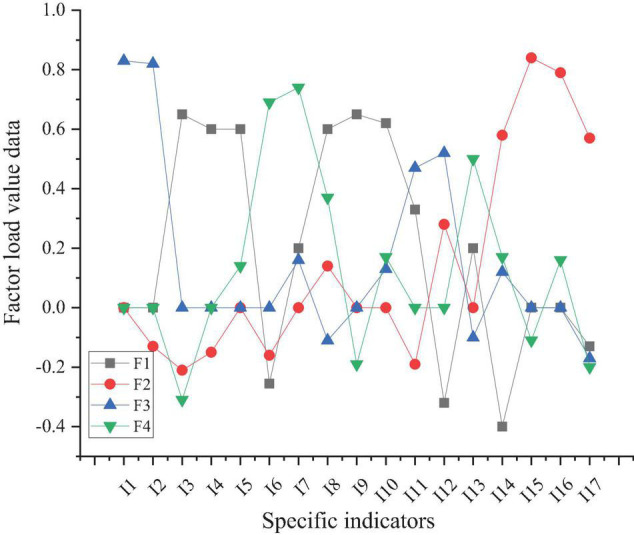
Results of the load value of the rotated factor (F1, F2, F3, F4 represent four principal component factors).

The load value of the rotated factor is shown in Figure 8.

Figure 8 shows that the specific indicators corresponding to factor 1 are ranked as I9, I3, I10, I4, I5, and I8 according to the load value. This indicator is added to the “music majors’ entrepreneurship policy” and the specific content of the indicator is family support, the government’s entrepreneurship policy, the local government’s support policy, friends who have started a business, school support, and the competitiveness of the entrepreneurial industry. The corresponding indicators of factor 2 are I12, I15, I16, I17, and the specific content is the hope to be recognized by friends, to achieve personal career success, to create wealth, and to be useful to society. This indicator is set to the column of “music majors’ entrepreneurial desire.” Factor 3 includes I1, I2, I11, and I12, which are related to the spirit of adventure and are named “the entrepreneurial quality of music majors.” Factor 4 includes I6, I7, and I13, which are related to practice, and it is set to “practice of entrepreneurship education for music majors.” From the above data, the survey results show that the greatest external support for music majors’ entrepreneurship comes from family support. A family’s political background, economic status, cultural heritage, degree of harmony, and the quality of family members are very important to a person’s growth and success. Good family environment, family support for entrepreneurship is an advantage of entrepreneurship, followed by government policy support. Government policy is based on the great convenience of entrepreneurs. Whether it is financial support or providing market, the guiding role of government policy is of great help to music majors’ entrepreneurship. In addition, according to the analysis of entrepreneurial psychological factors, “hope to be recognized by friends,” “achieve personal career success,” “create wealth” and “be useful to society,” these four factors are the psychological driving force of music majors’ entrepreneurship. The results also show that music related entrepreneurship training can cultivate students’ entrepreneurial quality and intention.

#### Strategy Optimization of Entrepreneurship Education for Music Majors

Through the factor analysis, four aspects of music majors’ entrepreneurship are obtained. The relevant strategies are proposed for the reform of entrepreneurship education and quality training of music majors. First, the general model of entrepreneurship education is introduced. The usual entrepreneurship education system includes entrepreneurship atmosphere, entrepreneurship environment, entrepreneurship courses, teachers, and entrepreneurship communication. The entrepreneurial system for students should arouse interest, improve entrepreneurial ability, and try to start a business, finally forming an entrepreneurial personality, which needs to be cultivated in the process of entrepreneurship. The subjects have certain advantages of entrepreneurial culture in the region, and the entrepreneurial environment is good. Under the influence of regional culture, music majors in the region have a strong enterprising spirit. The selected research university provides a good platform for music majors by creating a good external environment, offering entrepreneurship education courses following their aptitude, improving the quality of students, and promoting the advanced hardware facilities of entrepreneurship. The curriculum system of entrepreneurship education is also more suitable for the development of music majors, including the compulsory courses of entrepreneurship, corresponding lectures, and activity courses, which strengthen the education of case analysis. The most important part of the entrepreneurship education system is the construction of the practice system. Music majors accumulate the basic knowledge and practical skills of entrepreneurship through entrepreneurship practice activities, which lays a solid foundation for future entrepreneurship. The training of the teachers in the entrepreneurship education system is to employ teachers with entrepreneurial experience and provide students with more comprehensive entrepreneurial knowledge. The construction of the entrepreneur communication platform needs a fixed time and a fixed venue. Entrepreneurs can invite businessmen, and the number should be more than three. When entrepreneurs communicate with students, they mainly introduce entrepreneurial experience, exchange specialty, and solve students’ problems. The study area has a certain entrepreneurial fund support mechanism, which can provide financial support for music majors.

## Conclusion

Based on the severe employment situation, the reform and quality training of entrepreneurship education of music majors in colleges and universities. Based on introducing the background of entrepreneurship education, the advanced entrepreneurship education methods in foreign countries are summarized, the music majors in a university are selected to analyze the employment status, and finally, the education system suitable for music majors is proposed. The conclusion is that 22.2% of music students are very interested in entrepreneurship. In terms of music majors’ understanding of entrepreneurship policy, students with low understanding accounted for 40.1%. In the way that music majors acquire entrepreneurial knowledge, 8.00% of students usually choose books and networks. Music majors hope to get the support of the government and schools in cultivating entrepreneurship. In terms of entrepreneurship, 40.8% of music students intend to start a business according to their interests. The eigenvalue variances of the four main factors were 19.49, 12.96, 10.75, and 8.39%, respectively, and their contribution value was 51.58% The biggest external support for music majors’ entrepreneurship comes from family support, followed by policy support. In addition, the psychological driving factors supporting music majors’ entrepreneurship are “hope to be recognized by friends,” “obtain personal career success,” “create wealth” and “be useful to society.” This study provides a reference for entrepreneurship education in other universities. There are still some shortcomings in this study. For example, the elaboration of the entrepreneurship education system is not comprehensive. The follow-up research should study the entrepreneurship education system further.

## Data Availability Statement

The raw data supporting the conclusions of this article will be made available by the authors, without undue reservation.

## Ethics Statement

The studies involving human participants were reviewed and approved by the Shenyang Conservatory of Music Ethics Committee. The patients/participants provided their written informed consent to participate in this study. Written informed consent was obtained from the individual(s) for the publication of any potentially identifiable images or data included in this article.

## Author Contributions

The author confirms being the sole contributor of this work and has approved it for publication.

## Conflict of Interest

The author declares that the research was conducted in the absence of any commercial or financial relationships that could be construed as a potential conflict of interest.

## Publisher’s Note

All claims expressed in this article are solely those of the authors and do not necessarily represent those of their affiliated organizations, or those of the publisher, the editors and the reviewers. Any product that may be evaluated in this article, or claim that may be made by its manufacturer, is not guaranteed or endorsed by the publisher.
